# Excess volume addition method improves human resource efficiency and environmental sustainability of cytotoxic drug preparations

**DOI:** 10.1177/10781552251369431

**Published:** 2025-09-03

**Authors:** Marie Kroemer, Guillaume Galy, Severine Tarun-Coquoz, Camille Stampfli, Pauline Thomann, Antoine Pierrot, Laurent Carrez, Farshid Sadeghipour

**Affiliations:** 1 Department of Pharmacy, 30635 Centre Hospitalier Universitaire Vaudois, Lausanne, Switzerland; 2 Department of Informatics, 30635 Centre Hospitalier Universitaire Vaudois, Lausanne, Switzerland; 3 School of Pharmaceutical Sciences, University of Geneva, University of Lausanne, Geneva, Switzerland

**Keywords:** Antineoplastic agents, pharmaceutical services, drug compounding, sustainable development, pharmacy service, hospital, workflow

## Abstract

**Introduction:**

The increasing incidence of cancer entails a rising burden of cytotoxic drugs preparation. To improve the preparation process of cytotoxic drugs, specially designed half-filled bags with overfill capacity were manufactured and used in clinical routine. The aim of this study was to investigate the impact of using such bags on the duration of preparation, human resources, costs and environmental sustainability.

**Methods and Materials:**

A retrospective study comparing two methods, volume substitution and excess volume addition, for cytotoxic drug preparations was conducted over two periods of 6 months, within a University Hospital chemotherapy production unit. Volume substitution method (period 1; 5527 preparations) corresponded to the use of filled-bags. Excess volume addition method (period 2; 5108 preparations) corresponded to the use of half-filled bags. Preparation time included drug reconstitution and gravimetric controls. Data were extracted from the BD Cato^TM^ database.

**Results:**

Median duration of preparation using the excess volume addition method (2.4 min; IQR: 1.9–3.2) was significantly shorter than the volume substitution method (3.2 min; IQR: 2.6–4.1; *p* < 0.0001). It allowed saving 67 h during period 2, corresponding to 9.8% of a full-time equivalent technician. However, mean cost per preparation was significantly increased by 58% when using the excess volume addition method (p < 0.0001), due to higher costs of the newly designed bags. Broken down over the course of a year, the excess volume addition method would decrease the weight of cytotoxic waste for the entire hospital by 2.21%.

**Conclusion:**

Using the excess volume addition method with half-filled bags decreases time preparation, consumables and waste related to cytotoxic drug preparation.

## Introduction

Cytotoxic drugs preparation and handling is widely recognized as a high-risk activity.^1^ Centralization of cytotoxic drugs preparation, mainly parenteral cytotoxic drugs, offers the advantages of managing, controlling occupational exposure and securing their medication circuit.^2^ To help pharmacists and policies in elaborating a safe cytotoxic drugs medication circuit, the US National Institute for Occupational Safety and Health (NIOSH) provided a method to determine the hazardous potential of a drug as well as a continuously updated list of hazard drugs.^3^ The worldwide increasing incidence of cancer entails a rising burden of activity within centralized production unit of cytotoxic drugs preparation.^4,5^ In this context, improving the preparation process of cytotoxic drugs is necessary to promote human resource, economic and environmental sustainability.

To address this challenge, several approaches have been developed. One approach turned to technology for improving the production workload, through implementing a robotic compounder. Robots have been shown to increase staff safety and decrease some costs inherent to aseptic unit.^6,7^ Although literature indicate non-significant changes and even increases in drug preparation times following the implementation of robotic compounders, their ability to smooth preparation activities has been demonstrated.^6,8,9^ Another approach involved dose standardization of parenteral cytotoxic drugs, enabling the optimization of workload and work allocation streamlining preparation activities.^10–12^ By using robots or dose standardization, time required to prepare parenteral cytotoxic drugs remained at least constant. Reducing duration of preparation is a crucial point for streamlining preparation activities.

In the last decade, the assessment of environmental impact became a major concern for human activities, and this hold true for health. Although healthcare activities aim to improve peoples’ health, they also generate wastes that affect negatively the environment, and consecutively human health.^13^ Waste reduction from healthcare centers is a key environmental issue. With the emerging concept of “green hospital”, the 5Rs rule (reduce, reuse, recycle, rethink and research) is a suggested strategy to derive the maximum practical benefit while reducing waste.^14^ Among healthcare activities, operative rooms are one of the main consumer of single use disposable medical device and material and tend to be a good model for the implementation of sustainable measures whom waste reduction.^15^ Among technical platforms, centralized production unit within pharmacy departments are small units but they consistently generate waste related, among others, to the use of single-use sterile medical devices, cytotoxic drugs and personal protective equipment.

Traditionally, when preparing an injectable cytotoxic drug, the standard practice involves withdrawing a volume of diluent equal to the volume of the drug to maintain a constant final volume in the infusion bag. To reduce the duration of cytotoxic drug preparations, our aseptic unit implemented new bags with an overfill capacity to dilute active pharmaceutical ingredients, enabling us to avoid the preliminary step of withdrawing diluent. The aim of this study was to investigate the benefits from using these specially designed half-filled bags in terms of duration of preparation, human resources, costs and environmental sustainability.

## Material and methods

### Study design

This retrospective study was conducted at the production unit of the Department of Pharmacy of a 1546-beds University Hospital in Lausanne, Switzerland. The study was divided into two periods, from 20/10/2020 to 20/04/2021 (period 1) and from 21/04/2021 to 21/10/2021 (period 2), using two distinct procedures for cytotoxic drug preparations, to be detailed below. These periods were selected sequentially to reflect the real-world clinical implementation of a process change. Period 1 corresponded to routine practice using standard filled bags, while Period 2 began immediately after the introduction of half-filled bags with overfill capacity. Both periods were chosen to mitigate potential confounding factors such as seasonal variations or staffing fluctuations, as they were conducted under standard operating conditions with stable clinical demand. The procedures used for cytotoxic drug preparation during each period are detailed below.

### Cytotoxic drugs preparation

Cytotoxic drug preparations were performed in a biosafety cabinet (BSC, Cytobox CTB2-G, Steril, Milan, Italy) equipped with a computer using BD Cato^TM^ software interfaced with a label printer and a precision scale. Every step of the preparation was computer aided. Preparation time included the verification of each product (name, batch number and expiration date), the drug reconstitution (if necessary), the dilution steps of the cytotoxic with gravimetric controls, preparation, and infusion tubing filling (if necessary). Each volume either withdrawn or added was validated using BD Cato^TM^ software through the scale allowing the pursuit of the preparation (see supplementary method).

### Bags and procedures

During period 1 of the study, we used the “volume substitution method”. Specifically, the volume of diluent, equivalent to the volume of cytotoxic drug needed, was withdrawn using syringe(s). The technician used one or more syringes to withdraw the diluent before injecting the cytotoxic drug into the bag. Every syringe was single use and was discarded after use. All these steps are gravimetric controlled through the software BD Cato^TM^. Filled bags used were: NaCl 0.9% Ecobag 50, 250 and 500 mL and dextrose 5% Ecobag 250 mL (B:Braun, Melsungen, Deutschland)

During the period 2, we used the “excess volume addition method”. Specifically designed bags containing 0.9% NaCl or 5% dextrose and filled to half of their capacity/volume (named “half-filled”) were manufactured, by BBraun pharmaceutical^®^ team. The volume of cytotoxic drug needed was directly added to these bags, thus eliminating the withdrawing step and simplifying the preparation process. The excess volume addition method requires a re-evaluation of the total volume in which the cytotoxic drug is diluted. For instance, a bag with 500 mL volume/capacity was filled by BBraun pharmaceutical^®^ with 250 mL diluent instead of 500 mL. Half-filled bags used in this study were NaCl 0.9% Ecobag 50/100 mL, NaCl 0.9% Ecobag 250/500 mL, NaCl 0.9% Ecobag 500/1000 mL and dextrose 5% Ecobag 250/500 mL. The stability of drugs within these new volumes of diluent were validated through a review of the literature (Trissel guide, King Guide and Stabilis database) and manufacturer data.

### Data extraction

Data extracted from the BD Cato^TM^ database using a SQL request were: cytotoxic drug (active substance) volume (mL) and quantity (mg), final container type (e.g., bag, syringe or infusor), bag's volume, technician initials, medication number, preparation date, preparation starting and end time.

### Inclusion and exclusion criteria

Inclusion criteria were: cytotoxic drugs prepared in NaCl 0.9% or in dextrose 5% solutions within 250 or 500 mL filled/half-filled bags, cytotoxic drugs prepared in NaCl 0.9% within 50 mL filled/half-filled bags, cytotoxic drugs prepared as part of clinical trials, cytotoxic drugs needed volume superior or equal to 1 mL.

Exclusion criteria were: cytotoxic drugs prepared in syringe ready to be administered (bolus), cytotoxic drugs prepared in pump infusor, cytotoxic drugs prepared in non-half-filled bags (for instance 100 mL and 1000 mL).

### Assessment of saved time and costs

Comparative analyses of the “volume substitution” and “excess volume addition” methods were performed by calculating the time and cost of each preparation during period 1 and period 2, respectively. Preparation time, described above, was determined using the BD Cato™ software database. To determine human resources saved during each period, preparation time of all preparations included in the study were combined for each period (period 1 and period 2 respectively). Then, using the median duration of cytotoxic drug preparation during each period, human resources saved were estimated for respectively ten thousand and twenty-five thousand preparations. During the study period, half-filled bags were manufactured under contract justifying their high cost compared to standard filled bags (see supplementary method and supplementary Table 1). Costs calculated were those that provide variability according to the method of preparation used (volume substitution or excess volume addition). They included disposable materials (diluent bags and syringes) as well as technician direct labor costs at current regional standard hourly rates (29 €/hour in Canton de Vaud (2021), Switzerland).

### Environmental sustainability assessment

The number of syringes used was determined based on the total volume of cytotoxic drug needed during the two periods, obtained through data extraction from BD Cato^TM^ software. Based on data collected in 2021, we performed a simulation to determine the weight of syringes and diluent that would have been saved if the excess volume addition method had been used. Indeed, during period 2, there was no need to use syringe(s) to withdraw the diluent. Since the weight of cytotoxic waste produced by the production unit of the department of pharmacy was not available, this weight was deduced from the quantity of cytotoxic waste produced throughout the entire hospital.

### Statistical analysis

Statistical analyses were performed using GraphPad Prism 9 software (San Diego, United State). The level of significance was set at p < 0.05 for all tests and all tests were two-sided (* p ≤ 0.05, ** p ≤ 0.01, *** p ≤ 0.001 and **** p ≤ 0.0001). Continuous parameters were summarized with mean and standard deviation or median and interquartile range (IQR). Wilcoxon-Mann-Whiney test and unpaired t tests were used to compare mean or median value of continuous parameters.

## Results

### Use of excess volume addition method reduces the duration of preparation of cytotoxic drugs

**Figure 1.**
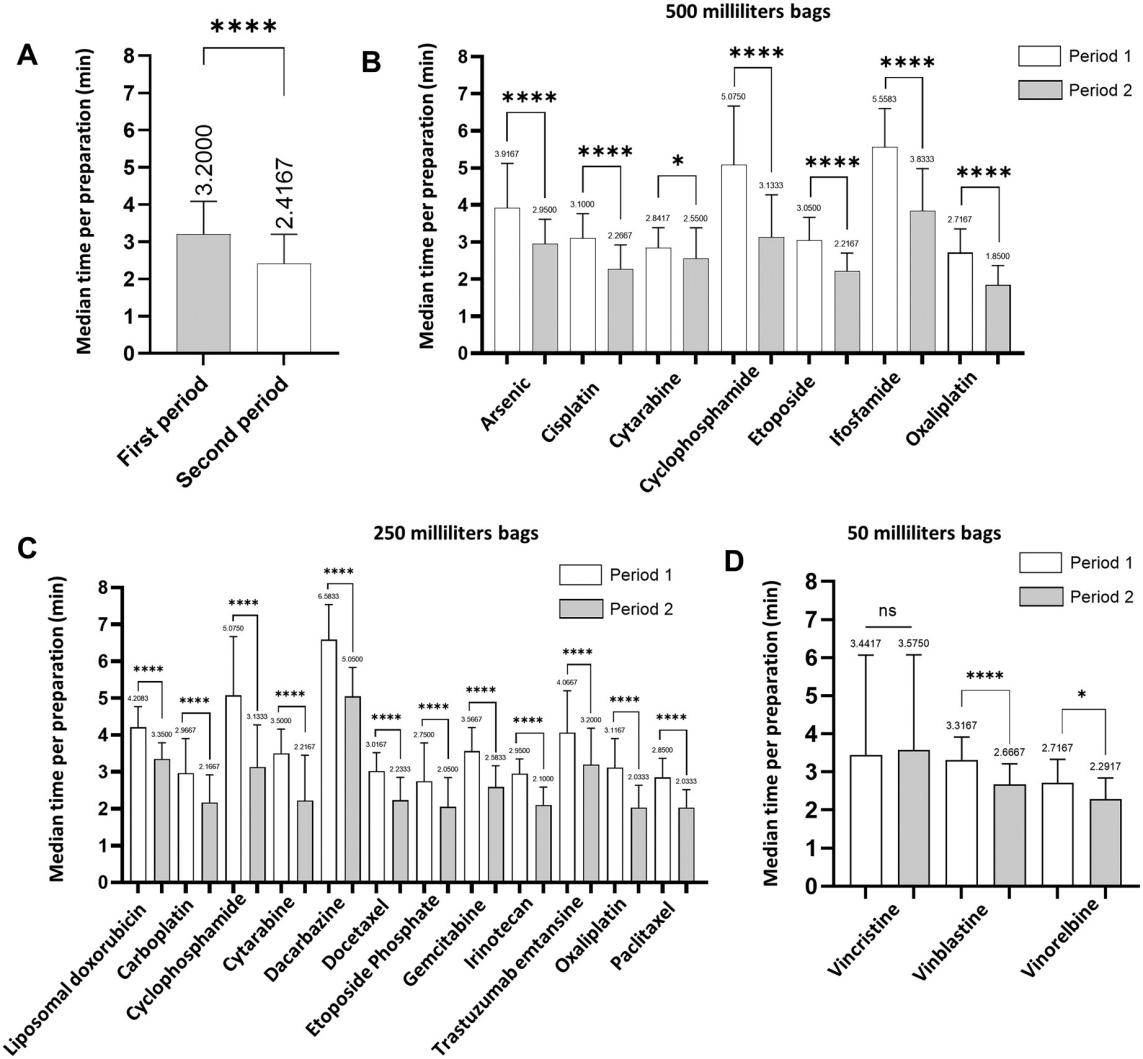
Use of excess volume addition method reduces the duration of preparation of cytotoxic drugs. A. Median cytotoxic drug preparation time during period 1 and period 2. B-D Median cytotoxic drug preparation time depending on active substance and the volume of the final container: (B) 500 mL, (C) 250 mL, and (D) 50 mL. Statistical differences were calculated using Mann-Whitney test or student t test. Median (IQR). *P < 0.05; ****p < 0.0001. ns, not significant.

Among the 10,635 cytotoxic drugs preparations that met the inclusion criteria during the study periods, 5527 (period 1) were prepared using the volume substitution method and 5108 (period 2) were prepared using the excess volume addition method. Overall, the median duration of cytotoxic drug preparation over the first period (3.2 min; IQR: 2.6–4.1) was significantly longer than in the second period (2.4 min; IQR: 1.9–3.2; *p* < 0.0001; Figure 1A). Based on median preparation time obtained over each period, we estimated that the excess volume addition method led to a reduction of 22.2% in manual preparation time by removing the diluent withdrawing step. We noticed that preparation's times varied depending on the type of cytotoxic drug (ready to use formulation or powder to be reconstituted), the volume of cytotoxic drug to dilute and handling instructions.

Median durations for cytotoxic drugs preparation were compared per active substance and volume of the final container (Figure 1B-D). For intermediate and larges volumes (respectively 250 and 500 mL), the excess volume addition method enabled to significantly decrease preparation times for all cytotoxic drugs (500 mL in Figure 1B and 250 mL *p* < 0.0001 in Figure 1C). For small volumes (50 mL), duration was significantly reduced for vinblastine (*p* < 0.0001) and navelbine (*p* = 0.024), but not vincristine (*p* = 0.3917; Figure 1D). It is noteworthy that vincristine exhibited the lowest median volume, estimated to 1.6 mL, across all periods, while other cytotoxic drugs consistently had a minimum volume of 4 mL. Next, we examined the inter-individual variability. The excess volume addition method significantly and consistently decreases median preparation duration, regardless of the technician (n = 8 technicians; Supplementary Figure 1A-F).

**Figure 2.**
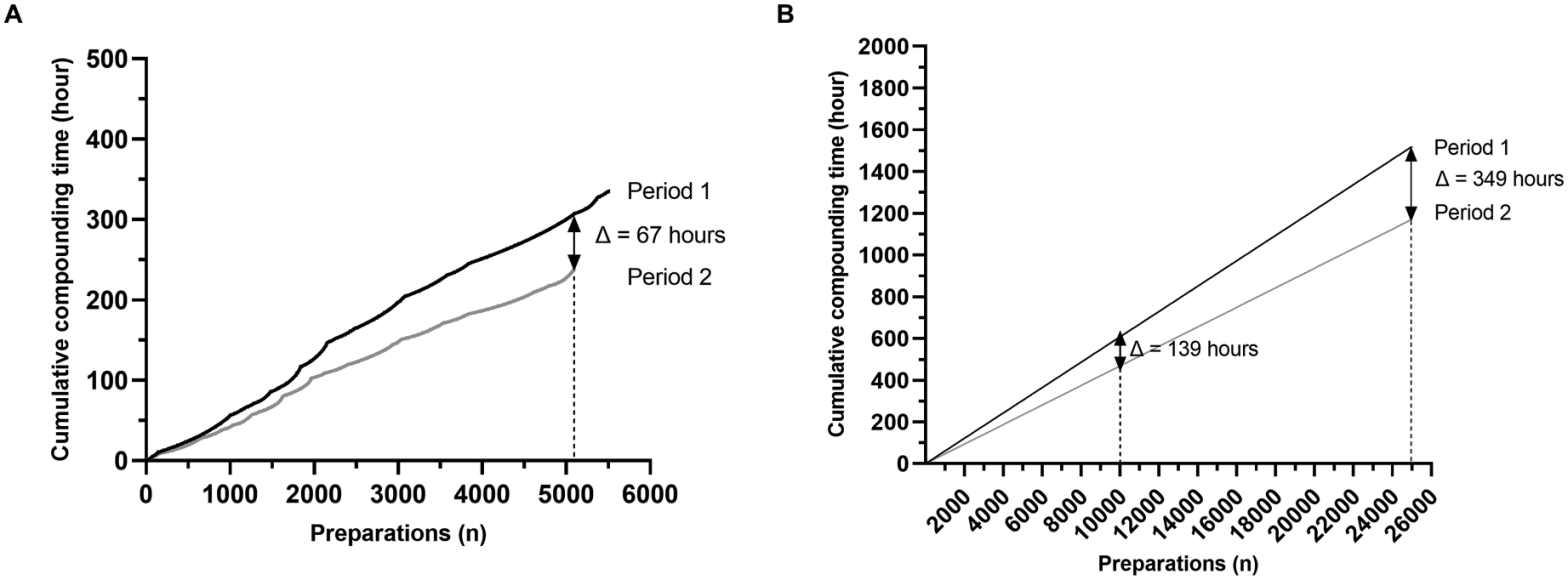
Use of excess volume addition method saves human resources. A. Added preparations’ times respectively for period 1 and period 2. B. Estimation by adding the median time of cytotoxic drug preparation during period 1 and period 2 of human resources saved for respectively ten thousand and twenty-five thousand preparations.

To assess the impact of the excess volume addition method on human resources, the total times of preparations were added respectively for period 1 and period 2 (Figure 2A). During period 2, new compounding modalities enabled saving 67 h in comparison to period 1, for an equal number of preparations (n = 5108). These 67 h corresponded to 9.8% of a full-time equivalent technician. Then, by adding the median duration of cytotoxic drug preparations during period 1 and 2, time saved was estimated for respectively 10,000 and 25,000 preparations (Figure 2B). Extrapolated to 10,000 and 25,000 preparations, the excess volume addition method would save 139 h and 349 h, respectively, in comparison to the volume substitution method. These 139 and 349 h equate to 20.3% and 51.1%, respectively, of a full-time equivalent technician.

### Use of excess volume addition method increases the costs of cytotoxic drugs preparations

**Figure 3.**
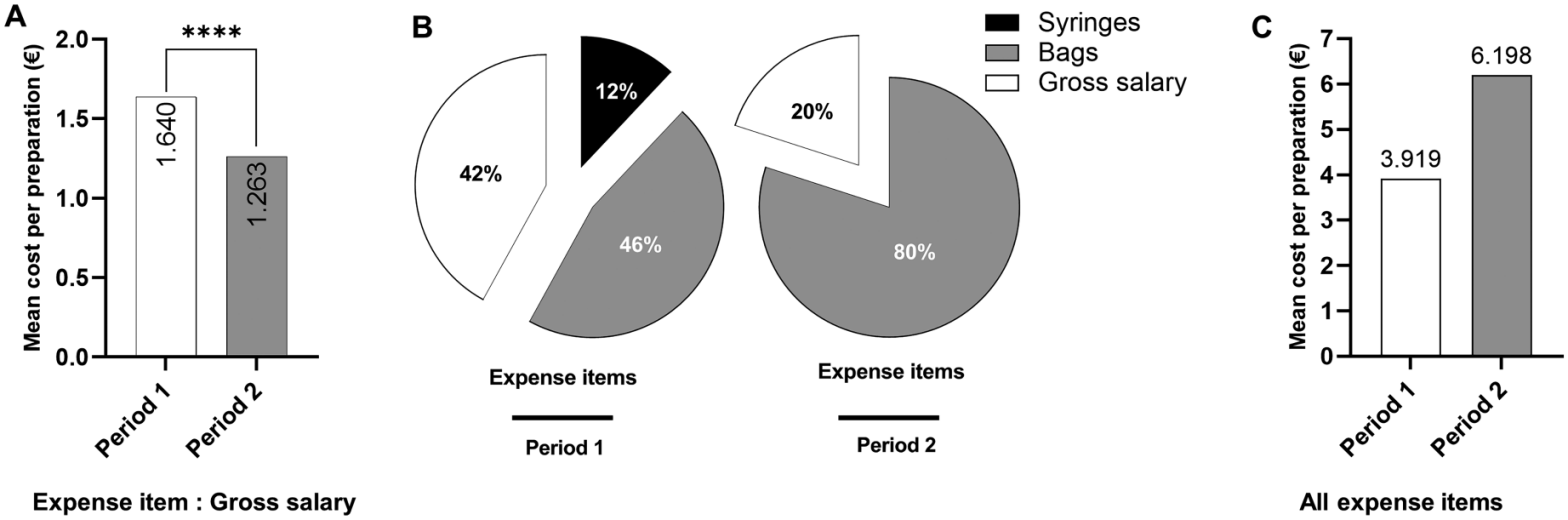
Use of excess volume addition method increases costs of cytotoxic drugs preparation. A. Mean cytotoxic drug preparation cost during period 1 and period 2 based on gross salary. B. Distribution of average expense items during period 1 (bag, syringe, gross salary) and period 2 (bag and gross salary). C. Mean cytotoxic drug preparation cost during period 1 and period 2 based on all expense items cited above.

First, we assessed the mean cost per preparation based on technician gross salary only. Considering this only expenditure item, the mean cost per preparation was significantly lower during period 2 (1.263 €) than during period 1 (1.640 €; p < 0.0001; Figure 3A). Next, all expense items including bag price, syringe price and technician gross salary were considered. During period 1, total costs were estimated to 21’660 € (3.919 € per preparation), of which gross salary was the main expense, representing 42% (Figure 3B). During period 2, total costs were estimated to 31’660 € (6.198 € per preparation), with bags being the main expense item, representing 80%. Overall, the mean cost per preparation during period 2 was increased by 58% when using a half-filled bag, corresponding to 10’000 € (Figure 3C). Extrapolated to 25,000 preparations, the excess volume addition method would cost 154’950 €, in comparison to the volume substitution method that would cost 97’975 €, representing an additional cost of 56’975 €.

### Use of excess volume addition method promotes healthcare sustainability

**Figure 4.**
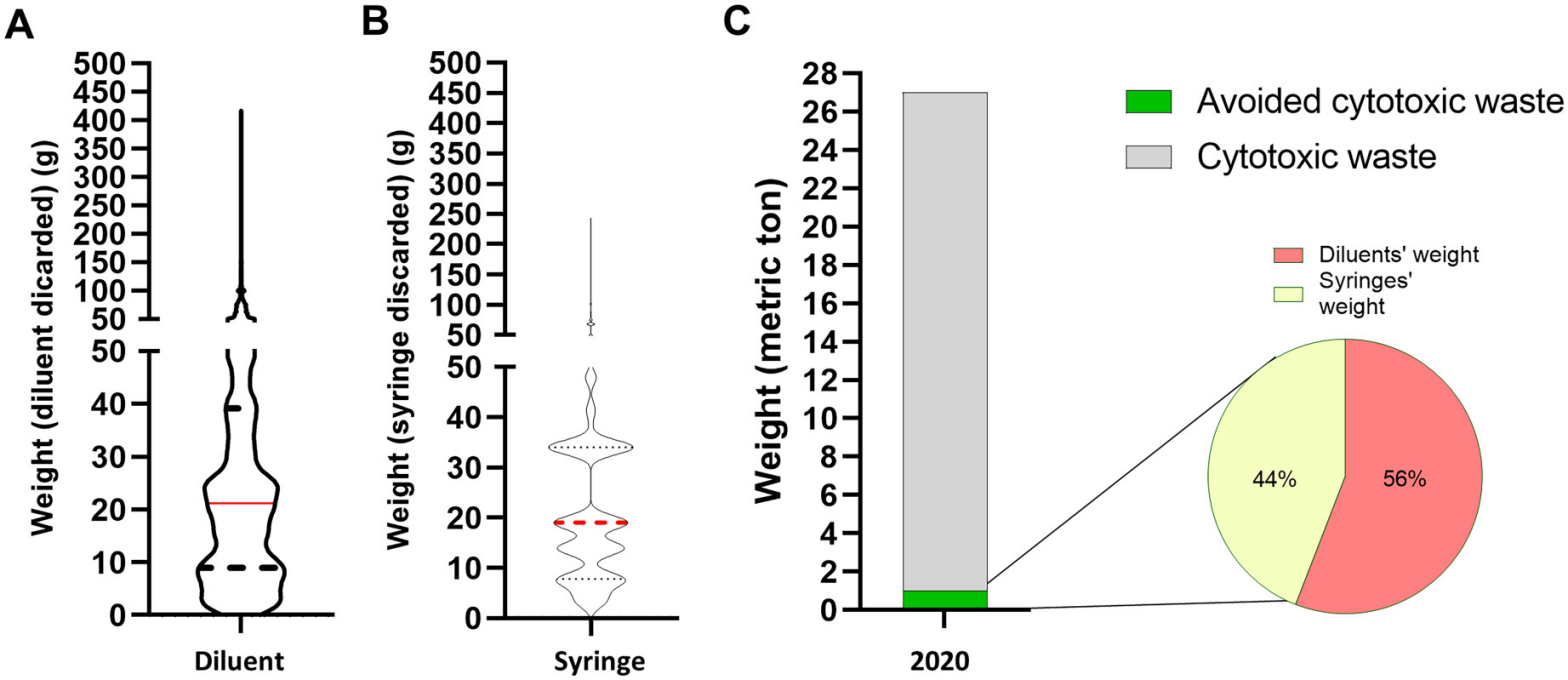
Use of excess volume addition method promotes healthcare sustainability. A. Median weight of diluent withdraws using the fixed volume method, 21.2 g (IQR: 8.9–39.2). B. Median weight of syringes used to take diluent discarded using the fixed volume method, 19.0 g (IQR: 7.8–34). C. Total weight of waste (syringes or diluents’ weight) that might be saved at the hospital level using the excess volume addition method (583 kg).

In 2021, 18,310 cytotoxic drugs were prepared in bags, of which 11,746 met the inclusion/exclusion criteria of the study. Considering these 11,746 preparations, median weight of diluent withdrawn was equal to 21.2 g (IQR: 8.9–39.2) and median weight of syringes used to remove discarded diluent was equal to 19.0 g (IQR: 7.8–34), bringing to a total weight of 583 kg (diluent: 329 kg; syringes: 254 kg) (Figure 4A and B). The weight of cytotoxic waste generated by the hospital in 2021 was equal to 26.24 metric tons. Hence, use of half-filled bags along 2021 is estimated to reduce the weight of hospital's cytotoxic waste by 2.21% by saving diluent withdrawn and syringes discarded (Figure 4C).

## Discussion

Rise of cancers’ incidence worldwide led to a continuous increase of the number of treated patients and workload in hospital day wards, directly impacting centralized production unit of cytotoxic drugs in hospital pharmacies. It is a daily challenge for pharmacists to optimize cytotoxic drug preparation's times to meet the expectations of oncology outpatient units. In the current study, we investigated the benefits of applying a pragmatic approach to reduce the time of cytotoxic drug preparation by using a newly and specifically designed half-filled bags, with overfill capacity. By deleting the diluent withdrawing step and enabling direct adding of cytotoxic drugs, our excess volume addition method allowed a significant reduction of the duration and costs of preparation of most cytotoxic drugs, while decreasing cytotoxic waste.

Although most solvent bags accept additions, their capacity is not sufficient to absorb large amounts of drug. To overcome this issue and address a need, we set-up a collaboration with a pharmaceutical group to specifically design and manufacture half-filled bags. These half-filled bags were manufactured under contract justifying their higher cost compared to filled bags. Consequently, the mean cost per preparation during period 2 was significantly higher than during period 1. So, we performed a simulation in which the price of half-filled bags was replaced by the one of filled bags. By reducing the price of half-filled bags to the price of filled bags the cost of each preparation was reduced and inferior during period 2 (equal to 2.944 €) in comparison to period 1 (equal to 3.919 €). Hence, large-scale production and marketing of half-filled bags would allow to save costs making our pragmatic approach cost-effective. As half-filled bags have proven to be of interest, our study illustrates the importance of close collaboration between hands-on health-care providers and the pharmaceutical industry to improve processes in an intelligent way.

In our study, the use of half-filled bags led to a reduction of 22.2% in manual preparation time by removing the diluent withdrawing step. As a result, our manual production process was simplified, and variances in withdrawal volumes were eliminated. Although the half-filled bags used in our study were manufactured under contract and may not be universally accessible, the method remains transferable; standard infusion bags typically contain an overfill volume, which, according to manufacturer instructions, can be safely used to dilute cytotoxic drugs, allowing institutions without contract-manufactured bags to adopt a similar strategy within validated safety margins. Other efficiency measures exist and could be implemented, such as batch preparation of standardized doses (also known as dose-banding) by the hospital pharmacy or industrially-manufactured ready-to-administer products, and the automated chemotherapy dose rounding rules of cytotoxic drug orders in the electronic health record.^16–18^ However, these methods do not directly modify the preparation steps, unlike our approach. Our approach should be considered in a broader perspective, as a complement to dose-banding and dose-rounding methods. Additionally, dose banding and dose rounding had other challenges, such as dealing with activities that require both standardized and personalized doses for patients. Production automatization is another strategy currently developed to optimize cytotoxic drug production process. The excess volume addition method could also enhance automate productivity by streamlining the preparation process. Our approach eliminates the convoluted tasks of withdrawing and injecting diluent, resulting in time savings for the machine.

Pharmacy technician, like other healthcare providers, often face repetitive hand and/or arm movements as well as prolonged periods of standing and/or walking. Repeating the same compounding motions during cytotoxic drugs preparation can be strenuous on the hands and wrists of pharmacy technicians.^19,20^ These repetitive motions and uncomfortable body postures are the primary contributors to work-related injuries.^21^ Withdrawing and injecting diluents and/or active compounds during product preparation are repetitive actions that require wrist rotation, pronation and supination when connecting and disconnecting medical devices, precision gestures and force when pulling or pushing the syringe plunger. These repetitive movements along a day can exacerbate musculoskeletal disorders. Therefore, reducing the number of repeated manipulations and gestures could contribute to decrease the risk of developing and/or aggravating musculoskeletal issues.^22^

Cytotoxic production units are heavy consumers of single use items. Some studies assessed strategies to reduce waste and medication wastage using dose rounding and standardized doses within chemotherapy production unit.^23,24^ From this point of view, our approach initially designed to reduced drug production time contributes to environmental sustainability, in accordance with 3 R out of 5 of the 5Rs rule (reduce, rethink and research) of the emerging concept of “green hospital”. Even though if the amount of waste recovered seems limited, our approach is simple and original. It highlights a simple lever for reducing the waste generated by hospital pharmacies. Although estimating the carbon footprint of syringes and diluent bags or the CO_2_ equivalent per unit weight for the incineration or treatment of hazardous medical waste, would have strengthened the sustainability assessment of our intervention, such an analysis was not feasible in the Swiss context at the time of the study due to the lack of publicly available life cycle data for medical consumables and detailed greenhouse gas emissions for the waste sector. By rethinking our production process, our collaboration with a pharmaceutical group enabled us to manufacture “better diluent bags” and demonstrates their benefits in terms of reducing drug preparation time, costs and waste. We are currently continuing this fruitful collaboration and working on the design of other half-filled bags capacity such as NaCl 0.9% 100/250 mL (100 mL in an empty 250 mL bag).

Our study has several limits (Table 1). Time spent for the logistical steps of picking, labeling, packaging and storage was not measured. Indeed, although logistical handling time was not measured, it was considered at least equivalent across both procedure for cytotoxic drugs preparation (see possibly lower with the excess volume addition method) and negligible in comparison to compounding time, based on our unit's standardized processes. We suspect that the use of half-filled bags would save time by reducing the required materials, in term of logistical, orders, storage and handling during the production. Additionally, only costs that provide variability were taken into account, unlike other studies that integrated all operating costs.^25,26^ Moreover, cytotoxic drug waste weight salvage was calculated for the whole establishment, not for the chemotherapy production unit because this information was not available. There is a risk of possible underestimation of cytotoxic drug waste salvage. Since actual cytotoxic waste weight per preparation was not directly measured, this global estimation may not fully reflect the reduction achieved within the pharmacy unit, where the use of half-filled bags directly reduced residual volumes and consumables. Finally, although a 7% increase in the proportion of powder to be reconstituted drugs was observed during Period 2 this did not affect production complexity, as lyophilized vial reconstitution was performed separately and was not included in the measured preparation time. Potential variations in formulation types were accounted for by stratifying preparation times by drug formulation and bag type in Figure 1B–D.

**Table 1.** Summary of study limitations, potential impacts, and mitigation strategies.

Limitation	Potential Impact	Mitigation Strategy
Logistical handling time not measured	Risk of underestimating time differences	Considered negligible and equivalent across both methods due to standardized workflow
Only variable costs included	May limit full cost assessment	Focused on cost components most sensitive to process changes
Waste salvage not specific to compounding unit	Potential underestimation of drug waste reduction	Acknowledged and discussed as a global estimation
No carbon footprint reduction estimation	reinforced the sustainability dimension of our findings	Not feasible due to lack of Swiss data; limitation acknowledged;
Drug mix changes between periods	Could confound time comparisons	Impact controlled by detailed analysis of preparation times by drug formulation and bag type to isolate effects. increase in powder to be reconstituted did not affect production time as reconstitution was performed separately and excluded from measurements

In conclusion, our excess volume addition method reduced the number of preparation steps by eliminating diluent withdrawing operations during cytotoxic drug preparation, ultimately reducing both the preparation time and number of manual steps required, while maintaining clinical efficacy and promoting environmental sustainability. This simple and pragmatic approach can be readily implemented in structures of any size.

## Supplemental Material

sj-docx-1-opp-10.1177_10781552251369431 - Supplemental material for Excess volume addition method improves human resource efficiency and environmental sustainability of cytotoxic drug preparations Supplemental material, sj-docx-1-opp-10.1177_10781552251369431 for Excess volume addition method improves human resource efficiency and environmental sustainability of cytotoxic drug preparations by Marie Kroemer, Guillaume Galy, Severine Tarun-Coquoz, Camille Stampfli, Pauline Thomann, Antoine Pierrot, Laurent Carrez and Farshid Sadeghipour in Journal of Oncology Pharmacy Practice

sj-tif-2-opp-10.1177_10781552251369431 - Supplemental material for Excess volume addition method improves human resource efficiency and environmental sustainability of cytotoxic drug preparations Supplemental material, sj-tif-2-opp-10.1177_10781552251369431 for Excess volume addition method improves human resource efficiency and environmental sustainability of cytotoxic drug preparations by Marie Kroemer, Guillaume Galy, Severine Tarun-Coquoz, Camille Stampfli, Pauline Thomann, Antoine Pierrot, Laurent Carrez and Farshid Sadeghipour in Journal of Oncology Pharmacy Practice

sj-docx-3-opp-10.1177_10781552251369431 - Supplemental material for Excess volume addition method improves human resource efficiency and environmental sustainability of cytotoxic drug preparations Supplemental material, sj-docx-3-opp-10.1177_10781552251369431 for Excess volume addition method improves human resource efficiency and environmental sustainability of cytotoxic drug preparations by Marie Kroemer, Guillaume Galy, Severine Tarun-Coquoz, Camille Stampfli, Pauline Thomann, Antoine Pierrot, Laurent Carrez and Farshid Sadeghipour in Journal of Oncology Pharmacy Practice
