# Supplementary material for: Excess volume addition method improves human resource efficiency and environmental sustainability of cytotoxic drug preparations
Source: J Oncol Pharm Pract. 2025 Sep 3;31(8):1329–37. doi: 10.1177/10781552251369431 (PMC12605280; doi:10.1177/10781552251369431)
Supplement: sj-docx-1-opp-10.1177_10781552251369431 - Supplemental material for Excess volume addition method improves human resource efficiency and environmental sustainability of cytotoxic drug preparations [file sj-docx-1-opp-10.1177_10781552251369431.docx]

**Supplementary material and methods**

**Cytotoxic drugs preparation**

The production unit complies with good manufacturing practices. Cytotoxic drug orders were fully computerized within the electronic health record (EHR, Soarian Clinicals, Oracle Cerner, Kansas City, United States) interfaced with a computerized gravimetric software (BD Cato™, Becton, Dickinson and Company, Franklin Lakes, United States). Before cytotoxic drug preparation, a hospital pharmacist reviewed and validated each order, according to a standardized pharmaceutical analysis process. Material needed for cytotoxic drug preparation were collected in a ready to use kit including a bag of NaCl 0.9% or dextrose 5%, luer-lock syringe(s), the undiluted cytotoxic drug (powder to reconstitute or ready to use solution), withdrawal and injection perforator (spike), infusion tubing and diluent vials (NaCl 0.9% or water for injection) if necessary. Cytotoxic drug preparations were performed in a biosafety cabinet (BSC, Cytobox CTB2-G, Steril, Milan, Italy) equipped with a computer using BD Cato^TM^ software interfaced with a label printer (ZEBRA ZD410-HC, Zebra Technologies, Lincolnshire, United State) and a precision scale (MS6002TS, Mettler Toledo, Greifensee, Switzerland). Every step of the preparation was computer-aided. The preparation was initiated by the technician within the BSC and the end of the preparation was marked by the printing of the label. Preparation time included the verification of each product (name, batch number and expiration date), the drug reconstitution (if necessary), the dilution steps of the cytotoxic with gravimetric controls, preparation, and infusion tubing filling (if necessary). Each volume either withdrawn or added was validated using BD Cato^TM^ software through the scale allowing the pursuit of the preparation.

**Assessment of saved time and costs**

Comparative analyses of the “Volume substitution” and “excess volume addition” methods were performed by calculating the time and cost of each preparation during period 1 and period 2, respectively. Preparation time, described above, was determined using the BD Cato™software database. To determine human resources saved during each period, preparation time of all preparations included in the study were combined for each period (period 1 and period 2 respectively). Then, using the median duration of cytotoxic drug preparation during each period, human resources saved were estimated for respectively ten thousand and twenty-five thousand preparations. During the study period, half-filled bags were manufactured under contract justifying their high cost compared to standard filled bags. Half-filled bags costs were equal to 4.1 € for NaCl 0.9% Ecobag 50/100 mL, 4.7 € for NaCl 0.9% or dextrose 5% Ecobag 250/500 mL, and 5.7 € for NaCl 0.9% or dextrose 5% Ecobag 500/1000 mL, filled bags costs were equal to 3.0 € for NaCl 0.9% Ecobag 50 mL, 1.7 € for NaCl 0.9% or dextrose 5% Ecobag 250 mL, and 1.9 € for NaCl 0.9% or dextrose 5% Ecobag 500 mL. Costs calculated were those that provide variability according to the method of preparation used (Volume substitution or excess volume addition). They included disposable materials (diluent bags and syringes) as well as technician direct labor costs at current regional standard hourly rates (29 €/hour in Canton de Vaud (2021), Switzerland).
