# Supplementary figures and images for: Excess volume addition method improves human resource efficiency and environmental sustainability of cytotoxic drug preparations
Source: J Oncol Pharm Pract. 2025 Sep 3;31(8):1329–37. doi: 10.1177/10781552251369431 (PMC12605280; doi:10.1177/10781552251369431)

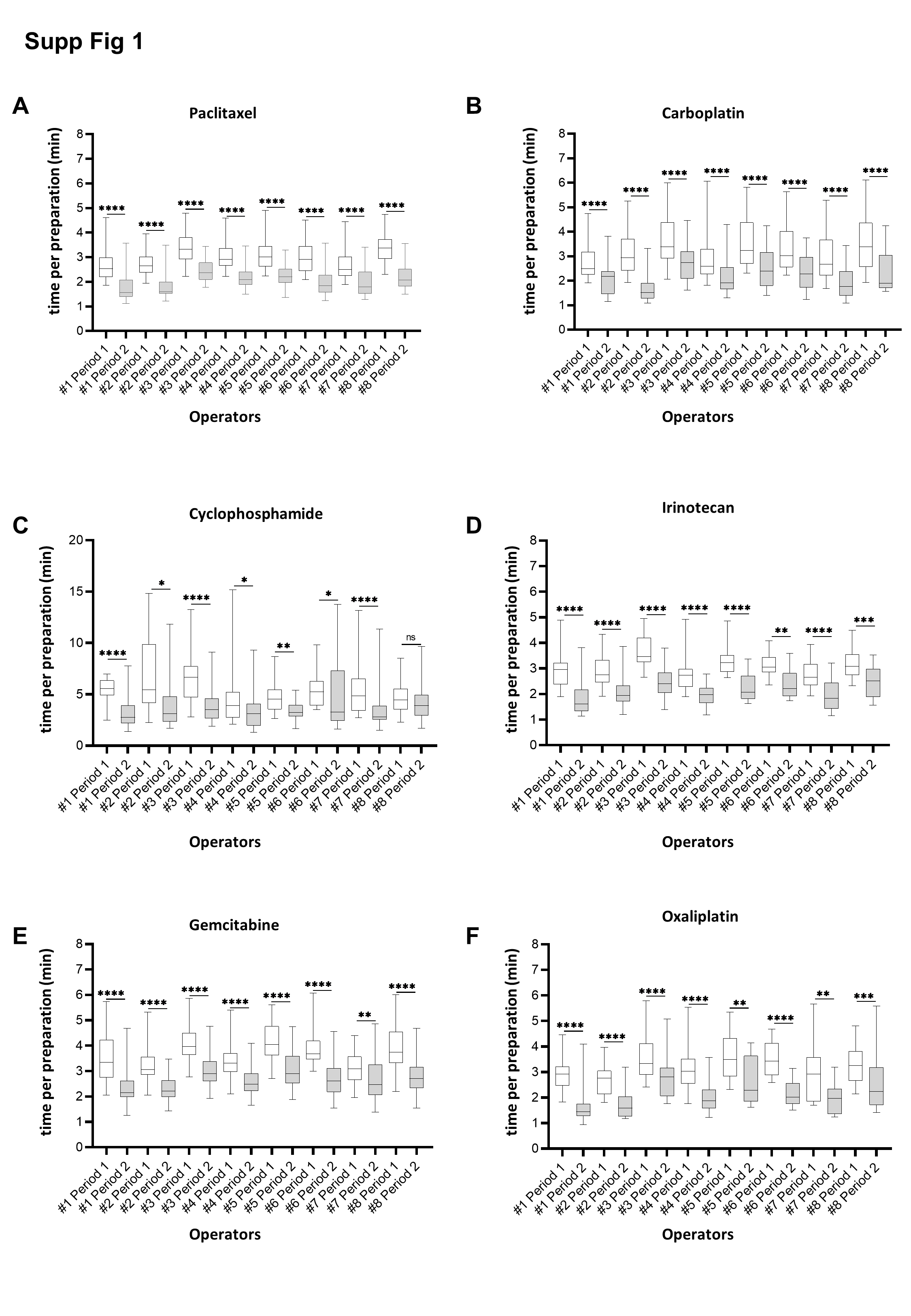

Supplement: sj-tif-2-opp-10.1177_10781552251369431 - Supplemental material for Excess volume addition method improves human resource efficiency and environmental sustainability of cytotoxic drug preparations [file sj-tif-2-opp-10.1177_10781552251369431.tif]
