# Supplementary material for: Excess volume addition method improves human resource efficiency and environmental sustainability of cytotoxic drug preparations
Source: J Oncol Pharm Pract. 2025 Sep 3;31(8):1329–37. doi: 10.1177/10781552251369431 (PMC12605280; doi:10.1177/10781552251369431)
Supplement: sj-docx-3-opp-10.1177_10781552251369431 - Supplemental material for Excess volume addition method improves human resource efficiency and environmental sustainability of cytotoxic drug preparations [file sj-docx-3-opp-10.1177_10781552251369431.docx]

**Supplementary Table I. Cost of filled and half-filled bags per volume.**

| Volume (mL) | Filled bags cost (€) | Volume (mL) | Half-filled bags cost (€) |
| --- | --- | --- | --- |
| 50 | 3.0 | **50/100** | 4.1 |
| 250 | 1.7 | **250/500** | 4.7 |
| 500 | 1.9 | **500/1000** | 5.7 |
